# Olfactory training with four and eight odors: comparison with clinical testing and olfactory bulb volumetrics

**DOI:** 10.1007/s00405-023-08283-4

**Published:** 2023-11-04

**Authors:** Sotiria Genetzaki, Vasilios Nikolaidis, Konstantinos Markou, Iordanis Konstantinidis

**Affiliations:** 2nd ORL Academic Department, Aristotle University, Thessaloniki, Greece

**Keywords:** Olfaction, Olfactory bulb, Plasticity, Regeneration, Training

## Abstract

**Purpose:**

Post-infectious olfactory dysfunction (PIOD) is one of the most common causes of olfactory impairment but has limited treatment options. Recently, olfactory training (OT) has been considered an effective treatment method; however, several questions have arisen regarding its optimal scheme. The aim of this study was to assess whether an OT scheme with 8 odors is more effective than the classic OT scheme with 4 odors by comparing psychophysical test results and olfactory bulb (OB) volumetrics.

**Methods:**

In this prospective cohort study, 72 patients with PIOD were included. The patients followed either the classic 4-odor OT scheme (COT; *n* = 34 patients) or an extended 8-odor scheme (EOT; *n* = 38 patients) for 16 weeks. All patients underwent olfactory testing with a Sniffin’Sticks battery test at 0, 8, and 16 weeks. Of the patients, 38 underwent brain magnetic resonance imaging for OB volumetric assessment before and after treatment.

**Results:**

The comparison of the olfactory test results did not show any significant difference between the two study groups, in agreement with the OB volumetrics. The convex OB showed better test results than the non-convex OB, with significantly better improvement after treatment regardless of OT type. The EOT group presented significantly better adherence than the COT group.

**Conclusion:**

The number of odors did not appear to play a significant role in the effect of the OT. However, the training scheme with more than four odors showed better adherence among the patients in a long-term treatment plan. The shape of the OB may have prognostic value in clinical assessment and warrants further investigation.

## Introduction

Post-infectious olfactory dysfunction (PIOD) is one of the most common causes of olfactory loss [1]. The viruses associated with PIOD include several types of rhinoviruses and the influenza virus, parainfluenza virus, Epstein–Barr virus, human immunodeficiency virus, and novel severe acute respiratory syndrome coronavirus 2 (SARS-CoV-2) [2, 3].

The subject of the present study was PIOD caused by viruses other than SARS-CoV-2, as our data were collected mainly before but continued during the COVID-19 (coronavirus disease) pandemic. However, given the common features of SARS-CoV-2 olfactory dysfunction (OD), our study outcomes can also be applied in its management [4].

The most commonly recommended treatment option for PIOD is olfactory training (OT), relying on the unique plasticity of the olfactory system and the safety, efficiency, and low cost of the treatment [5]. Most studies on patients with PIOD have been conducted using the initial 4-odor scheme, as suggested by Hummel et al. [6]. A relatively small number of studies have examined different training schemes. Altundag et al. achieved better results using a modified OT (MOT) scheme where patients used three sets of four odors sequentially than the classic scheme [7]. Other studies used five and six odors by chance or four odors with different qualities, presenting mixed results [8–10]. A recent multi-centric study showed no significant difference in clinical outcome between a 4- and an 8-odor treatment scheme in patients with COVID-19 [11]. However, the odors were selected randomly, without categorisation. Recent studies have shown an even greater efficacy of OT combined with medications in long-term olfactory dysfunction due to COVID-19 infection [12]. However, questions regarding its optimal scheme still need scientific answers. Within this frame, the aim of the present study was to clarify whether the use of eight instead of four odors in OT offers better outcomes and patient adherence.

## Materials and methods

### Patients

Our study included 72 patients with a history of PIOD (range 6–13.5 months). The patients’ mean age was 43.4 ± 4.3 years (range 21–55 years). The exclusion criteria were age ≤ 18 years, pregnancy, and OD-causing disorders, such as neurodegenerative diseases and sinonasal, post-traumatic, congenital, and idiopathic olfactory dysfunctions. To keep our results more homogeneous, we also excluded patients who developed OD after SARS-CoV-2 infection, as the patient recruitment started before the COVID-19 (coronavirus disease) pandemic.

After providing a detailed medical history, each patient underwent a full ENT (ear, nose, and throat) examination. Patients with endoscopic findings of chronic rhinosinusitis were also excluded from the study.

Olfactory testing was performed at 0.8 and 16 weeks. Furthermore, all patients were given the option to undergo magnetic resonance imaging (MRI) of the olfactory bulb (OB) at 0 and 16 weeks after treatment. All patients reported having qualitative symptoms, such as parosmia and phantosmia.

This study obtained written informed consent from all the patients and was conducted in accordance with the Declaration of Helsinki for Medical Research Involving Human Subjects and approved by the ethics review committee of Aristotle University of Thessaloniki (9201/22-7-2017).

### Olfactory testing

Olfactory function was assessed using the Sniffin’Sticks battery test (Burghart GmbH, Wedel, Germany). The phenyl ethyl alcohol odor threshold (*T*) was calculated using the staircase method with 16 dilutions at ascending concentrations. The examiner continued toward lower concentrations when the odor was correctly identified in two successive trials or toward higher concentrations when the odor was not recognized in one trial. The total number of reversals was 7, and the threshold was defined as the mean of the last 4 staircase reversals (score range 0–16). Odor discrimination (*D*; score range 0–16) and identification (*I*;score range, 0–16) were evaluated. The total threshold/discrimination/identification (TDI) score was calculated by adding all subsets (score range 0–48) for each patient. On the basis of the results, the patients were characterized as normosmic (TDI > 30.5), hyposmic (16.5–30.5), and functionally anosmic (< 16.5). An increase in TDI score of ≥ 6 can be considered a clinically significant improvement of olfactory function [13].

### Classic OT

As described by Hummel et al. [6], the procedure included exposure to four odors twice a day for 5 min over 16 weeks. The four odors (rose: phenyl ethyl alcohol, eucalyptus: eucalyptol, lemon: citronellal, and cloves: eugenol) were carefully selected as representatives of the four main odor categories defined by Henning [14].

Each 5-min session requires an alternated exposure of around 10 s to each odorant, with a 10-s interval between odorants. The patients were encouraged to perform OT at a fixed timing every morning and evening and to keep a written diary of all sessions. Those who did not adhere to the treatment for > 7 days were excluded from the study.

### Extended OT

Our extended OT (EOT) scheme was based on the categorical dimensions of human odor descriptor space by Castro et al. [15]. Castro et al. applied non-negative matrix factorisation to Dravniek’s odor profile database and derived 10 fundamental odor qualities: (1) fragrant, (2) woody/resinous, (3) fruity (non-citrus), (4) chemical, (5) minty/peppermint, (6) sweet, (7) smoky, (8) lemon, and two sickening odors: (9) pungent and (10) decayed. We excluded the sickening odor categories to achieve optimum patient compliance with the training. The EOT consisted of eight odors in the same order as the odor qualities: (1) rose: phenyl ethyl alcohol; (2) eucalyptus: eucalyptol, (3) banana: methyl butyl acetate, (4) turpentine: terebinthine, (5) mint: menthol, (6) vanilla: vanillin, (7) almond: benzaldehyde, and (8) lemon: citronella. The patients followed the scheme twice a day for 16 weeks, as in the classic scheme.

### Study groups

The present study clarified whether EOT compared with COT would improve the efficacy of OT and whether OT adherence would differ between the groups. After a comprehensive discussion of the possible benefits of OT, each patient was given the option to choose either the COT or the EOT. The COT group (*n* = 34) followed a 4-odor olfactory scheme, and the EOT group (*n* = 38) followed an 8-odor olfactory scheme.

MRI for OB volumetric assessment before and after treatment was offered to all the patients; however, only 18 and 20 patients in the COT and EOT groups, respectively, presented in both MRI sessions and included in our analysis.

### MRI volumetric evaluation

OB volumetric evaluations were performed with a 3-Tesla MRI system (Siemens, Erlangen, Germany) with coronal T2-weighted fast spin-echo images that included the anterior and the middle parts of the skull base. The scanning parameters were as follows: repetition time/echo time, 4800/152 ms; slice thickness, 1 mm; matrix size, 256 × 256; average, 2; in-plane resolution, 0.4 × 0.4 mm; and no intersection gap.

The OB volume was measured using manual segmentation with an AMIRA 3D visualization and modeling system (Visage Imaging, Carlsbad, USA). The contours of the left and the right OBs were manually delineated. The proximal limit of the OB was determined on the basis of the sudden change in the diameter at the beginning of the olfactory tract. After all surfaces were added and multiplied by the slice thickness, the volume in cubic millimeters was obtained for the right and the left OBs, and the total OB volume was calculated as the sum of the two values.

Measurements were performed blindly twice by two examiners (IK and SG). When the difference in volume was > 10%, the examiners performed a third measurement together. The mean measurements were included in the database.

In addition, the shape of the OB morphology was assessed on coronal T2images and categorized as convex (oval, olive, or plano-convex-shaped) and non-convex (banana, plane, irregular, or scattered) according to the classification by Yan et al. [16].

### Statistical analysis

Data were analyzed using SPSS 20.0 (SPSS Inc., Chicago, IL). Demographics, Sniffin’Sticks test results, and OB volumetrics are reported as mean ± standard deviation or percentage (%).Unpaired *t* test and Pearson correlation coefficient were used to compare the study groups. Bonferroni adjustment was used for post hoc analysis. The alpha level was set at *p* = 0.05.

## Results

**Fig. 1:**
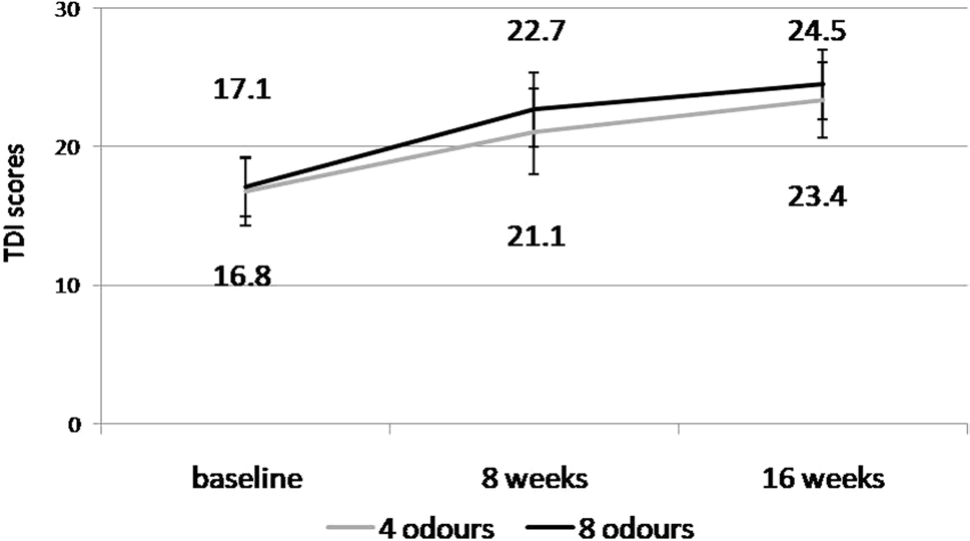
Comparison of olfactory test results between the two study groups before and after olfactory training

No significant differences in age, sex, and the incidence of parosmia were found between the study groups (*p* = 0.42, *p* = 0.64, and *p* = 0.32, respectively). Overall, OT showed a positive effect on olfaction, as indicated by the TDI scores (*F*_1,72_ = 10.44, *p* = 0.005). The TDI scores significantly improved in both groups from baseline to 16 weeks (COT: *p* = 0.0031, EOT: *p* = 0.002) but did not significantly differ between the groups during the study period (Fig. 1). The absolute number of patients who showed clinical improvement did not significantly differ between groups (EOT: 21/38 [55.2%] vs. COT: 18/34 [52.9%]).

The comparison of ΟΒ volumetrics between the groups was in agreement with that of TDI scores, without significant differences (Table 1). The overall OB size (in both groups) increased as expected after OT, with a trend toward significance (mean total OB: baseline, 82.7 ± 14.8 mm^3^; post-OT: 92.9 ± 10.2 mm^3^, *p* = 0.071). The OBs of the patients with parosmia presented slightly lower volumes, but the difference between the groups was not significant (Table 1). None of the patients reported phantosmia.

**Table 1:** OB volume measurements before and after treatment and significance of differences (asterisk indicates statistical significance)

	Baseline	16 weeks	Sign (*p* value)
COT			
Right OB	41.34 ± 7.5 mm^3^	46.58 ± 6.9 mm^3^	0.075
Left OB	40.5 ± 8.8 mm^3^	45.9 ± 7 mm^3^	0.067
Total OB	81.67 ± 16.5 mm^3^	92.3 ± 13.5 mm^3^	0.068
EOT			
Right OB	42.12 ± 6.2 mm^3^	47.25 ± 6.5 mm^3^	0.081
Left OB	41.43 ± 7.8 mm^3^	46.1 ± 8.1 mm^3^	0.095
Total OB	83.61 ± 13.5 mm^3^	93.45 ± 7.5 mm^3^	0.086
Convex OB	44.1 ± 7.1 mm^3^	50.3 ± 7.8 mm^3^	0.032*
Non-convex OB	39.2 ± 8.8 mm^3^	41.4 ± 8.1 mm^3^	0.21
Parosmia OB	40.81 ± 7.3 mm^3^	44.7 ± 6.9 mm^3^	0.12
Non-parosmia OB	42.92 ± 7.8 mm^3^	47.3 ± 8.1 mm^3^	0.101

* the significant difference of convex OBs volume before and after OT

**Fig. 2:**
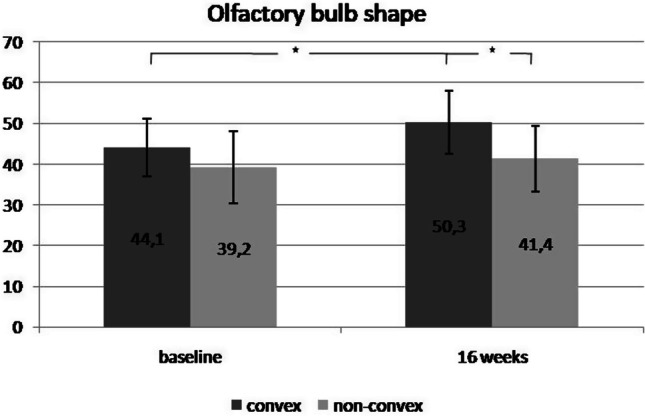
Comparison of convex and non-convex OB volume at baseline and 16 weeks later. The two asterisks indicate first (from left to right) the significant increase of convex OBs size in 16 weeks time and the second the significant difference between convex and non-convex OB size at 16 weeks assessment

The study groups had similar convex and non-convex OB shape distributions, with most patients having non-convex shapes (60.5%). The convex OBs showed higher values before and after treatment than the non-convex OBs, regardless of OT scheme (Fig. 2). The mean increases in the numbers of convex and non-convex OBs were 14.05% and 5.61%, respectively (*p* = 0.025).

**Fig. 3:**
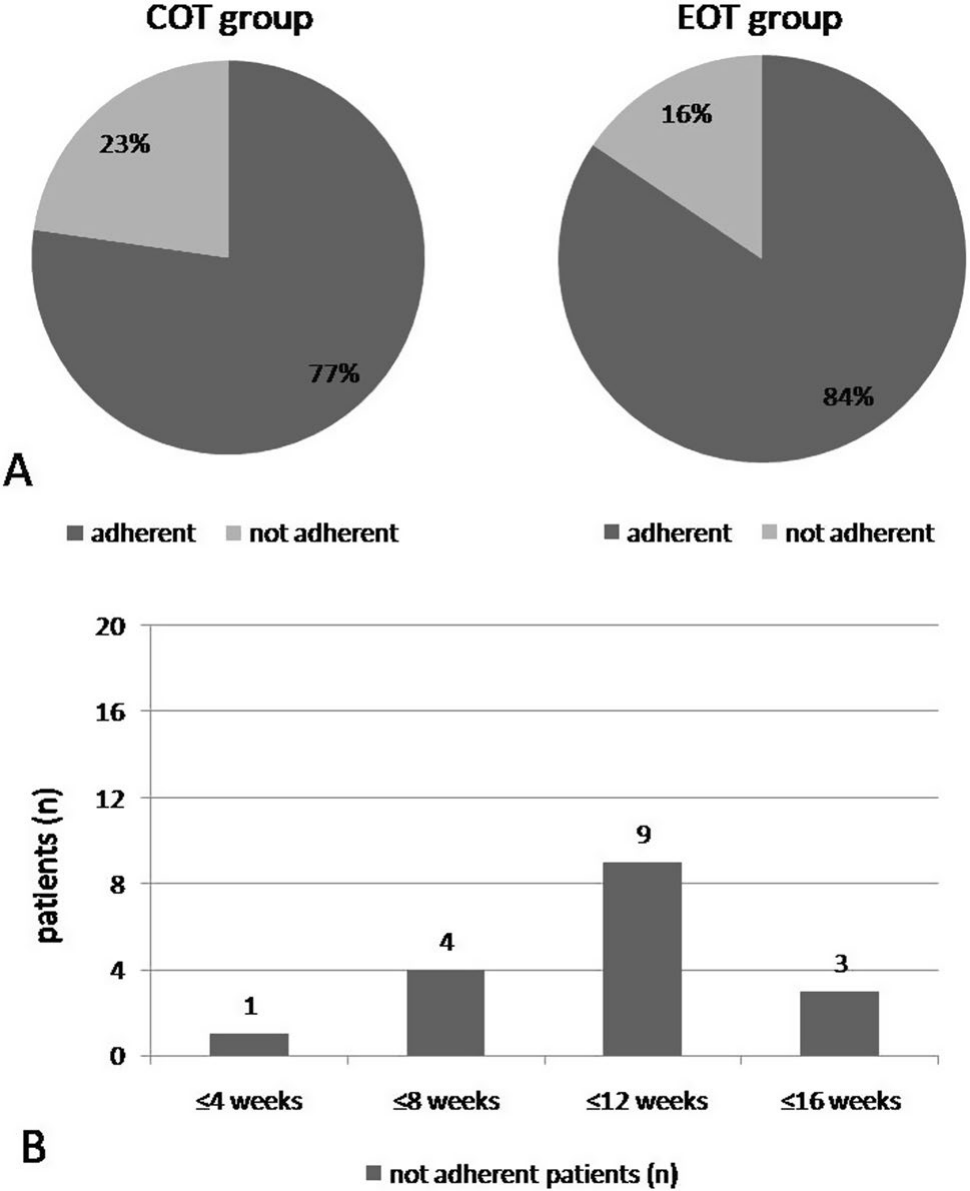
**A** percentages of patients' adherence in OT from both study groups. **B** Absolute number of patients and their timing of OT discontinuation. The majority of patients who did not completed the OT period can be seen to discontinue the scheme between 8-12 weeks of OT

The patients in the EOT group showed significantly better adherence to the COT (Fig. 3A). Seventeen patients discontinued both OT schemes, mostly between 8 and 12 weeks of OT, regardless of OT type (Fig. 3B). Fifteen of the 17 patients reported a non-positive effect on olfaction as the reason for their decision.

The TDI results moderately correlated with the total OB volumes at baseline and after treatment (baseline: *r* = 0.42 and *p* = 0.004, post-treatment: *r* = 0.38 and *p* = 0.021).

## Discussion

OT is part of the current PIOD treatment and supported by many controlled trials and meta-analyses [17, 18]. However, improvement in olfactory function was reported in only 50–65% of patients [18]. Various studies have tried to improve the effectiveness of OT using odors, concentrations, and durations different from those in the COT scheme, without convincing results [5].

The present study has three major conclusions:

1. The use of eight odors did not further improve the COT outcomes based on clinical testing and OB volumetrics.
2. Patient adherence to the 8-odor OT scheme was better.
3. Convex OB volumetrics showed higher values before and after treatment, regardless of training scheme.

Stimulation of the olfactory system with more odor categories was the basic idea behind our study, based on evidence of the spatial organization of the olfactory system at the levels of the OB and central brain regions [19, 20]. Thus, if the brain odor space is not occupied homogenously but in a clustered manner, different perceptual qualities could lead to different OT outcomes. However, this was not the case in the clinical setting, as demonstrated in our study. This is in agreement with other studies that suggested that the molecular characteristics of odorants used in OT have limited effect on olfactory outcome [11, 21].

Altundag et al. concluded that periodical alteration of the odors used during OT improved the treatment success rate [7]. By contrast, Oleszkiewicz et al. found no significant difference in outcomes between COT with mixed odors and odor-altering OT, suggesting that OT outcomes are not strongly influenced by the training regimen [21]. In a multi-centric randomized clinical trial of 80 patients with persistent OD after COVID-19 infection, Pires et al. used 8 odors, without better results than the COT [11]. Other authors presented promising results from altering-odor OT but did not compare these with those of the COT scheme [22].

Compliance with the OT scheme is still a significant problem in everyday clinical practice, as physicians should propose a regimen interesting enough to be followed in the long term and to inform patients that olfactory progress depends on their regular engagement. Within this frame, a more complex OT scheme could be more interesting for patients and thus achieve better adherence. In our study, adherence was significantly better in the 8-odor group, similarly to other studies that used more complex OT schemes than COT [9, 22].

Most patients who resigned from our protocol quit OT at a period of 8–12 weeks, regardless of OT type and reported no positive effect on olfaction as the main reason for their decision. This finding may be used by physicians to enhance the internal motivation of patients to adhere to the training protocol by formulating communications focused on this period.

Our OB measurements confirmed the clinical test results, showing that OT had the same positive effect on OB size in both groups. OB volumetrics is a reliable and objective method for assessing olfactory function, with a significant prognostic value for recovery [23]. Our study has the particularity of presenting OB data before and after OT. Only a few studies have presented such data in patients with sinonasal disease and post-laryngectomy OD [24, 25]. OB volume showed a relatively quick response to the16-week OT scheme and was higher in the convex-shaped OBs.

MRI is not part of the standard clinical assessment for patients with PIOD in most countries. However, the fact that not only the volume but also the shape of the OB may play a role in the prognosis of OD raises the question of whether it should be included in the diagnostic workup. Our results are in conjunction with the study by Yan et al. [16] that indicated that a convex OB shape is associated with better olfactory outcome. In addition, the authors suggested that certain shapes correlated with specific causes of olfactory disorders. For example, an irregular OB shape was often observed in post-traumatic olfactory loss. This evidence suggests that OB shape can be used as a biomarker for OD.

The limitations of our study include the absence of a control group, which ideally should receive placebo OT with odorless substances, and the relatively small number of patients included in the OB measurements. An open trial always has the weakness of non-blind allocation concealment as this causes bias related to the subjects’ knowledge of their assignment, which might make them more or less likely to drop out of the trial, leading to missing data. However, the use of objective methods for outcome assessment and blind examiners, as we did for the OB measurements, can reduce this bias.

The optimal OT scheme is still a field of scientific discussion, mainly due to its fast introduction as a treatment option, and should be further investigated before we explore the mechanisms of OT effectiveness with basic science studies on molecular and cellular levels.

## Conclusion

In this study, OT for 16 weeks with 8 odors did not provide a better outcome than the classic 4 odors, adding evidence that the OT regimen cannot influence OT outcomes. However, it appeared to present better patient adherence. The two study groups showed similar OB measurements and clinical testing results. The shape of the OB may play a role in treatment success as the convex OBs presented better outcomes before and after treatment, regardless of the type of OT used.

## Abbreviations

PIOD: Post-infectious olfactory dysfunction
OD: Olfactory dysfunction
OT: Olfactory training
COT: Classic olfactory training
EOT: Extended olfactory training
OB: Olfactory bulb
